# Global trends in measles publications

**DOI:** 10.11604/pamj.supp.2020.35.1.18508

**Published:** 2020-02-20

**Authors:** Rachel Kornbluh, Robert Davis

**Affiliations:** 1 Yale University, New Haven, Connecticut, USA; 2 American Red Cross, Nairobi, Kenya

**Keywords:** Measles, epidemiology, surveillance, eradication, elimination, coverage, panencephalis (SSPE), routine immunization, supplementary immunization activities (SIAs)

## Abstract

**Introduction:**

Beginning with the 1960s, this review analyzes trends in publications on measles indexed by the National Library of Medicine from January 1960 to mid-2018. It notes both the growth in numbers of published papers, and the increasing number and proportion of publications, in the current century, of articles on such items as costing, measles elimination, and determinants of coverage.

**Methods:**

A two-person team extracted from the National Library of Medicine (NLM) homepage all citations on measles beginning in 1960 and continuing through mid-2018. These were then classified both by overall number and by subject matter, with tabular summaries of both by decade and by subject matter. The tabular presentation forms the basis for a discussion of the ten most frequently cited subjects, and publication trends, with a special emphasis on the current century.

**Results:**

As in the past, the most often currently published items have been on coverage and its determinants, measles elimination, outbreak reports, SSPE, and SIAs. The putative relationship between vaccination and autism saw a spurt of articles in the 1990s, rapidly declining after the IOM report rejecting the causative hypothesis.

**Conclusion:**

There is a discussion on the sequencing of polio and measles eradication, the former unlikely before 2022, and an examination of likely research priorities as the world moves from measles control to measles eradication. There is a key role for social science in combatting vaccination reticence. The role of technical innovations, such as micropatch vaccination, is discussed.

## Introduction

The licensing of the monovalent measles vaccine (now joined by combination vaccines, including MR and MMR) led, in the developing countries, to its inclusion, in the 1960s, in routine immunization programs. The 1974 creation of the Expanded Programme on Immunization extended the reach of measles immunization to the developing world. The growth in program development has been matched by a concomitant growth in published articles on measles (Table 1). We sought, in the present article, to review both major topics of interests to authors, decade by decade, and trends in publishing (Table 2).

**Table 1:** Published citations on measles by decade

	1960-1969	1970-1979	1980-1989	1990-1999	2000-2009	2010 – mid-2018
**Number of Articles Published**	2434	3629	3907	4894	5455	5762

Source: www.pubmed.gov, retrieved on 1 July 2018

**Table 2:** Top 12 most-published topics of the present decade, with ranking in prior decades

	1960-69	1970-79	1980-89	1990-99	2000-09	2010-18
Rank						
1	Subacute sclerosing panencephalitis, SSPE, 26	Measles epidemiology, 34	Outbreak reports, 41	Vaccination coverage, including determinants, 121	Autism, 166	Vaccination coverage, including determinants, 266
2	Measles epidemiology, 21	SSPE, 21	Measles elimination, 39	Outbreak reports, 82	Vaccination coverage, including determinants, 164	Measles elimination, 220
3	Measles eradication, 19	Supplementary Immunization Activities, 19	Measles epidemiology, 34	Measles elimination, 67	Outbreak reports, 153	Outbreak reports, 218
4	Supplementary Immunization Activities, 11	Outbreak reports, 13	Measles eradication, 31	Supplementary immunization activities, 62	Measles elimination, 120	SSPE, 146
5	Progress in measles control & elimination, 5	Measles eradication, 11	Studies of costing and economics, 23	Measles epidemiology, 54	Supplementary Immunization Activities, 70	Supplementary Immunization Activities, 84
6	Routine immunization, 4	Studies of costing and economics, 11	Vaccination coverage, including determinants 22	Measles eradication, 47	Progress in measles control and elimination, 62	Progress in measles control and elimination, 77
7	Measles elimination, 2	Mathematical modeling of measles, 4	SSPE, 17	Mathematical modeling of measles, 41	Measles epidemiology, 54	Mathematical modeling of measles, 74
8	Outbreak reports, 2	Progress in measles control and elimination, 3	Mathematical modeling of measles, 13	Autism, 32	Mathematical modeling of measles, 48	Routine immunization, 72
9	Studies of costing and economics, 1	Measles elimination, 3	Supplementary immunization activities, 12	Studies of costing and economics, 24	Measles eradication, 36	Measles epidemiology, 68
10	Vaccination coverage, including determinants, 0	Routine immunization, 3	Progress in measles control and elimination, 8	Progress in measles control and elimination, 22	Studies of costing and economics, 30	Studies of costing and economics, 67
11	Mathematical modeling of measles, 0	Vaccination coverage, including determinants, 1	Routine immunization, 7	Routine immunization, 12	Routine immunization, 29	autism, 50
12	Autism, 0	Autism, 0	Autism, 0	SSPE, 12	SSPE, 9	Measles eradication, 50

Citation counts for each decade are in commas after the name of the topic.

## Methods

This paper presents findings on trends in measles publications using decade-by-decade frequency distribution analyses of key topics addressed in the published literature from 1960 to mid-2018. To obtain the frequency analyses presented, articles indexed as measles by the National Library of Medicine database were divided by decade from the 1960s to the 2000s. In order to capture current trends in publication, articles published from January 1st, 2010 to June 30th, 2018 were also examined as a partially-completed decade.

A list of 98 unique keywords was developed, and keywords were grouped into 61 relevant topics (e.g., the terms supplementary, supplemental, and campaign were used to identify articles discussing supplementary immunization activities). Counts were then taken of the number of articles per decade that contained each keyword in their titles. Only articles that employed the pertinent definition of a keyword were included. Topic counts were subsequently obtained by summing the count of each keyword assigned to a topic, taking into consideration any duplicates that might occur from a title containing multiple keywords assigned to the same topic.

Once this analysis was completed, the top twelve most frequently published topics of the present decade were identified. These topics were tracked and arranged in a data table that ranks their frequency in each decade (Table 2). A discussion follows on trends in published discussion of these twelve topics.

The table above shows the number of citations by decade. Table 2, more detailed, shows the frequency distribution of topics by decade.

### Limitations/caveats

While this paper presents trends in publications on measles literature, it includes only publications from the National Library of Medicine database; gray literature and other databases were not analyzed. As a result, this paper cannot capture trends in all measles literature (whether published or unpublished), nor can it definitively reveal trends across all instances of published measles literature. At times, topic analyses were limited by this; thus, some references that were not identified through the search method described above were included in topic analyses.

In its exploration of trends on publications in measles literature, this paper used a Boolean search of keywords in titles of articles related to measles. As a result, published papers that did not list keywords directly in their titles were excluded from topic counts, even if the keywords were featured centrally in their analyses. Guidelines were created to establish when the context of a keyword was grounds for inclusion in a topic count. Each keyword was examined by a single reviewer to increase consistency in inclusion. Despite these measures, some amount of error in interpretation and subjectivity likely affected topic counts..

## Results

### Topic analyses

Vaccination coverage, including determinants: the importance of coverage

In recent years, published citations on measles vaccination coverage have outnumbered those on all other topics, even measles elimination, with 266 citations on coverage from 2010 to mid-2018 in the indexed measles literature.

High vaccination coverage, with one dose or (more recently) two doses of measles containing vaccine, forms the basis for all progress in measles vaccination and for the eventual eradication of the disease. Calculating coverage permits governments and partners to track progress towards meeting the targets of the *Global Vaccine Action Plan 2011-2020* [1] as well as the *Global Measles and Rubella Strategic Plan* [2].

A variety of methods for gauging vaccination coverage have superseded the classic 30 x 7 cluster coverage methodology, which was used from the 1970s well into the present century [3]. The revised WHO guidelines, published in June 2018, enable survey planners more flexibility in making adjustments for confidence intervals and other survey parameters [4].

The 1998 creation of the joint reporting form, now submitted by 192 governments, has permitted WHO and UNICEF to publish, in the *Weekly Epidemiological Record and the Morbidity and Mortality Weekly Report,* increasingly refined estimates of coverage at national, regional and global levels. The article Global Routine Vaccination Coverage, 2016, published in the *MMWR,* provides the most recent published WHO/UNICEF estimates of coverage by antigen and dose. In the words of the article,

“MCV1 coverage in 2016 ranged from 72% in the African Region to 96% in the Western Pacific Region and from 20% to 99% by country. During 2015-2016, MCV1 coverage has remained stable or increased in all regions. Globally, 123 (63%) countries achieved the GVAP 2020 target of 90% national MCV1 coverage [5].

The importance of MCV2 vaccination is that, without it, there is a failure rate of 15 percent among those vaccinated once at 9 months of age. For 2016, according to the *MMWR,*

“MCV2 coverage by WHO region varied from 24% (African Region) to 93% (Western Pacific Region), including countries that have not yet introduced MCV2. In four of six WHO regions (African, Region of the Americas, Eastern Mediterranean, and South-East Asia), MCV2 coverage increased in 2016 compared with 2015, because of both an increase in coverage in multiple countries, as well as an increase in the number of countries introducing MCV2. Globally, MCV1 coverage was 85% and MCV2 coverage was 64% in 2016 (estimated dropout = 21%) [5].

These data show high coverage in some regions, but by no means all. They have led to much work, and to much research work, seeking ways to raise routine vaccination coverage.

Of the six WHO regions, the African region, with 47 member states, lags the others in coverage. However, a 2007 publication noted incremental improvements in vaccination coverage in the African region, largely financed, in low income countries, from external sources [6]. In the current decade, coverage in most African countries has plateaued.

#### Research and publication on coverage determinants

Many authors have analyzed the determinants of vaccination coverage with a view to increasing coverage. Cochrane reviews and other systematic reviews have sought to summarize the voluminous existing literature on methods for improving routine vaccination coverage [7, 8].

Of course, no efforts at improving coverage can be measured without good data. The early decades of EPI, which was launched in 1974, have few citations on coverage, perhaps because reliable data were generally lacking in the early years of the programme.

By contrast, the current century has been rich in publications on coverage and coverage innovations. For routine coverage, national vaccination registers have been established and documented in Brazil, Israel and Norway. These registers are useful in tracking progress of individual patients in a highly mobile population and a check on the accuracy of parent retained vaccination records. As of this writing, in late 2018, electronic immunization registers are being evaluated in, among other countries, Tanzania and Zambia.

Routine reporting, whether in traditional or electronic form, cannot be accepted without surveys. Most developing countries are the subject of periodic national surveys, such as Demographic and Health Surveys, which serve as a check against administrative coverage estimates, i.e., those estimates which are based on the application of doses given to best demographic estimates of the target population. In addition to the DHS methodologies, the current century has brought about application to coverage monitoring of such methods as LQAS (lot quality assurance surveys), independent monitoring, and cluster coverage surveys, for which the methodology was recently revised by WHO [4, 9].

In the current century, the data quality audit method (DQA) and the less costly data quality self-audit (DQS) have permitted GAVI and governments correctly to gauge the extent to which national administrative figures correspond to the data gathered at the field level [10].

In some countries, national coverage surveys are done in tandem with EPI programme reviews, or preceding them, so that the survey data can feed into recommended corrective measures.

The present century has also seen the introduction of equity analysis in order to ascertain coverage by socio-economic status. It has seen publications on such topics as *“Monitoring equity in vaccination coverage: A systematic analysis of demographic and health surveys from 45 Gavi-supported countries [11].”*

With coverage data now disaggregated both by geography and by socio-economic status, planners can take corrective measures to raise coverage in underserved localities and in underserved socio-economic groups.

Although this century has seen methodological advances in coverage analysis, there is one area which remains under-researched: the coverage status of adults. The following citation is an exception to the general rule of analyzing mainly the coverage status of infants and children: *Why are young adults affected? Estimating measles vaccination coverage in 20-34 year old Germans in order to verify progress towards measles elimination [12].”*

Especially in Europe, where adult measles is emerging as a potential obstacle to regional elimination, new methods, or adaptations of old ones, may be necessary as a step towards elimination.

#### Measles elimination: elimination and eradication of measles disease

The related topics of elimination and eradication have been among the most discussed in the measles literature. Discussions of elimination and eradication started in the ‘60s, soon after the 1963 licensing of the vaccine. From 21 citations on these topics in the ‘60s, citations have soared to 270 in the period since 2010.

In discussions on these related topics, it is well to remember that eradication refers to interruption of transmission on a global scale, with permanent reduction of incidence to zero. Elimination, by contrast, refers to interruption of transmission at the regional or national levels, with the possibility of virus reintroduction from endemic areas [13].

##### The 20th century

Although David Morley asked as early as 1969 whether measles eradication was possible [14], it was only in 1982 that Hopkins and colleagues published *The Case for Global Measles Eradication”,* the first formal advocacy article for global eradication to appear in the indexed literature [15].

In the early decades of measles vaccination, the world was focused on the eradication of smallpox, with eradication declared by the World Health Assembly only in 1980. Resources were not then available for measles eradication. With the 1980 declaration of smallpox eradication, some authors turned to the question *of What next? in terms of feasibility.* The benchmarks of eradicability were such criteria as the absence of a non-human reservoir, the availability of safe and effective vaccines, the presence of political will and financial support, and adequate surveillance and laboratory resources to track progress towards eradication.

The 1980s saw many articles from North America and Europe (especially Czechoslovakia and the Scandinavian countries) on national elimination efforts. Starting in the 1990s, the Pan American Health Organization ran a series of articles, especially in its *EPI Newsletter,* on national elimination efforts in Latin America, and on PAHO/CDC partnership for regional elimination. In the 1990s, PAHO´s *EPI Newsletter* published 38 articles on measles elimination and related topics.

Broadly speaking, the 20th century debates centered on whether measles eradication was feasible. The 21st century debates, noting the elimination of measles from the Americas, have focused on what preconditions must be met before the virus can be cleared from all six WHO regions.

##### The 21st century

EPI marked four milestones about the turn of the century: the 1998 creation of the Joint Reporting Form, a first step in creating uniform databases shared by W.H.O. and UNICEF; the 2001 creation of the Measles Initiative (now the Measles and Rubella Initiative); the 2002 elimination of measles from the Americas; and the creation, in the new century, of the Global Measles Laboratory Network, an indispensable adjunct to case-based measles surveillance. In the new century, there have been dozens of articles on the status of national and regional elimination efforts, especially from the Western Pacific, the European Region, and the Region of the Americas.

In addition to these milestones, the UN Millennium Development Goal 4 called for steep declines in under-five mortality by 2015 in comparison to 1995 baselines. MDG4 focused attention on measles mortality reduction as a major tool in reaching under-five mortality reduction goals [16].

The Junior Research Fellowship (JRF), which led to improved reporting on coverage and incidence, was a move in the right direction. So, too, was the creation of the Measles Initiative (now the Measles & Rubella Initiative), an international alliance to move forward the measles agenda. However, it was the clearance of measles from the western hemisphere, proof of concept on a continental scale, which lent credibility to the arguments of the eradication advocates. After 2002, published discussions on measles eradication shifted from “whether” to “how.” In particular, certain authors laid down prerequisites for the commitment to a global eradication effort, including Heymann and colleagues. Christie and Gay rejected the view that high routine immunization be a prerequisite for measles campaigns or a measles eradication goal [17].

By the year 2000, a group of Center for disease control (CDC) authors was ready to reaffirm the case made by Hopkins and colleagues in 1982. Their reasoning, which reflects that of most eradication advocates, is summarized in their abstract:

Measles eradication would avert the current annual 1 million deaths and save the $1.5 billion in treatment and prevention costs due to measles in perpetuity. The authors evaluate the biological feasibility of eradicating measles according to 4 criteria: (1) the role of humans in maintaining transmission, (2) the availability of accurate diagnostic tests, (3) the existence of effective vaccines, and (4) the need to demonstrate elimination of measles from a large geographic area. Recent successes in interrupting measles transmission in the United States, most other countries in the Western Hemisphere, and selected countries in other regions provide evidence for the feasibility of global eradication. Potential impediments to eradication include (1) lack of political will in some industrialized countries, (2) transmission among adults, (3) increasing urbanization and population density, (4) the HIV epidemic, (5) waning immunity and the possibility of transmission from subclinical cases, and (6) risk of unsafe injections [18].

The support structures for global eradication grew in the current century. Featherstone and colleagues wrote in 2003 on the development of the Global Measles Laboratory Network (GMLN), modelled on the global polio lab network [19].

The GMLN has served, in the current century, to complement case-based surveillance of measles and will serve, in future years, to assist in documenting measles elimination. Both case-based surveillance and the GMLN are essential complements to global eradication.

In the new century, articles appeared on such topics as Future Savings from Measles Eradication in Industrialized Countries [20]. Since measles is no longer a major cause of death in the developed world, such articles buttress arguments in favor of co-financing global eradication on the grounds of self-interest. More recently, Durrheim and Crowcroft have written on “The price of delaying measles eradication,” a price already being paid by the countries of the Americas, which must finance sustained regional elimination while waiting for global eradication [21]. These authors noted especially the problem of measles in those aged 15 years and over, a problem which will grow with each passing year as eradication is delayed.

In a widely quoted call to Go Big and Go Fast, S. B. Omer and colleagues noted the build-up of parental vaccine refusals amid declining incidence [22]. They stated that “If a disease such as measles is considered a priority by the global public health community, human and financial resources should be committed up front to a full-scale eradication initiative, conducted with a sense of urgency. If we don’t ‘go big and go fast,´ we may have to spend a prolonged period on eradication efforts with a diminished likelihood of success.”

The debate between integrated and vertical approaches to measles eradication has led to a 2017 call by Goodson and colleagues for a “diagonal approach,” with a better balance of integrated and mass campaign approaches than seen in GPEI [23].

In a recent review [24], Hinman listed the following issues in regard to measles: 1) Failure to meet Global Vaccine Action Plan (GVAP) goals; 2) Incomplete implementation of the Global Measles and Rubella Strategic Plan 2012-2020; 3) The GPEI transition, which presents both threats and opportunities; 4) GAVI transition/graduation - many of the same countries affected by GPEI transition are also going through transition/graduation from GAVI support, which entails gradual increases in country co-financing of vaccines up to fully self-financing; 5) Donor fatigue - we are now 17?years late in delivering on polio eradication and donors are increasingly vocal about wanting GPEI to complete so they can address other issues. Many are not enthusiastic about another eradication initiative; 6) Increase in nationalism/isolationism leading to reduced interest in many countries to support issues viewed as primarily affecting developing countries; 7) Need to integrate services - there is now less enthusiasm for categorical programmes and increasing demand for integrated health system development/strengthening.

##### Midterm review

The most significant recent publication on eradication is the midterm review of the Measles and Rubella Global Strategic Plan, by Orenstein and colleagues, published in 2018 in Vaccine [25]. Key highlights from their review: 1) Measles eradication is the ultimate goal but it is premature to set a date for its accomplishment. Existing regional elimination goals should be vigorously pursued to enable setting a global target by 2020. 2) The basic strategic approaches articulated in the Global Measles and Rubella Strategic Plan 2012-2020 are valid to achieve the goals but have not been fully implemented (or not appropriately adapted to local situations). 3) The report recommends a shift from primary reliance on supplementary immunization activities (SIAs) to assure two doses of measles-containing vaccine (MCV) are delivered to the target population to primary reliance on ongoing services to assure administration of two doses of MCV. Regular high quality SIAs will still be necessary while ongoing services are being strengthened. 4) The report recommends a shift from primary reliance on coverage to measure progress to incorporating disease incidence as a major indicator. 5) The report recommends that the measles/rubella vaccination program be considered an indicator for the quality of the overall immunization program and that measles/rubella incidence and measles and rubella vaccination coverage be considered as primary indicators of immunization program performance. 6) Polio transition presents both risks and opportunities: risks should be minimized and opportunities maximized. 7) A school entry immunization check could contribute significantly to strengthening overall immunization services with assurance that recommended doses of measles and rubella vaccines as well as other vaccines have been delivered and providing those vaccines at that time if the child is un- or undervaccinated. 8) Program decisions should increasingly be based on good quality data and appropriate analysis. 9) The incorporation of rubella vaccination into the immunization program needs to be accelerated - it should be accorded equivalent emphasis as measles. 10) Outbreak investigation and response are critical but the most important thing is to prevent outbreaks.

##### The elephant in the room of measles eradication

The year 2000 marked the target year for polio eradication, transmission of which persists as of this writing (2018) in three endemic countries (Afghanistan, Pakistan, and Nigeria). The tardy eradication of polio from the world has placed on hold early efforts to shift gears towards measles eradication, since the same governments and donors financing GPEI could not be expected simultaneously to launch a global push for measles eradication. As of 2018, GPEI has set a new target year of 2022 for completing its work. Only after 2022 is it likely that the global community can turn its undivided attention towards eradication of measles or measles and rubella.

The twin problems of increasing measles incidence in adults and increasing vaccine hesitancy amid declining childhood incidence argue in favor of a brief, highly financed push, lasting years rather than decades. This is the “big and fast” approach, in the words of Omer and colleagues [22].

#### Measles epidemiology, surveillance, and outbreak reports

The related areas of epidemiology, surveillance and measles outbreak investigation have all figured in the medical literature published since the ‘60s. Measles epidemiology, mostly descriptive, has figured in the literature since the ‘60s. Remarkably, surveillance and outbreak investigation articles appeared but rarely in the published literature on measles in the decade after the 1963 licensing of the vaccine.

In the 1970s, articles began to appear, especially from North America and the Soviet Union, on outbreak investigations. That decade also saw the appearance of articles on the seroepidemiology of measles. A growing number of authors now advocated for community serosurveys as a tool for planning the age range for measles campaigns [26]. The use of oral fluids has figured in recent literature [27].

It was perhaps the case investigations of highly vaccinated populations that led to the US government´s decision in favor of a two-dose regime, which is now global W.H.O. policy. The 1980s saw a stream of articles, continuing to the present, on the epidemiology of measles at the national and subnational levels, initially from North America, Africa, and South Asia. In the 1980s, after two decades of continuing endemic transmission with the one-dose policy, the problem of persistent measles transmission in vaccinated children was identified. Then, the United States adopted, in 1989, a two-dose vaccination regime, following the example of New York State, which was the first in the U.S. to do so [28]. The internal dialogue among New York decision makers is recounted by Orenstein (op. cit.).

“A small meeting in New York State broke the log jam on moving to a routine 2-dose schedule. College outbreaks in the state captured the attention of the Health Commissioner, David Axelrod. He called together academic infectious disease specialists, led by Saul Krugman and Martha Lepow, state and county health officials and representatives of the CDC to decide how best to address the problem. During the meeting, consultants agreed that the major problem with measles in colleges was failure to make an adequate immune response after a single dose of measles vaccine rather than waning immunity. Led by Saul Krugman, the academic pediatric infectious disease experts had already come to the conclusion that a second dose of measles vaccine would be necessary if measles elimination was the goal. However, the public sector representatives resisted, primarily because of cost considerations. After spirited discussion, the group did not reach unanimity about whether to recommend a routine 2-dose schedule. Near the end of the meeting, Dr. Axelrod came in to hear the conclusions and said emphatically, don´t tell me what it costs, tell me what is the right thing to do.” He pointed out that New York State should be preventing outbreaks, not trying to control them, and declared that New York State would implement a 2-dose schedule even if it were the only state. Public sector opposition to a 2-dose schedule rapidly melted.”

Subsequently, the World Health Organization recommended two doses of measles-containing vaccine. By 2008, the two-dose regime was policy in 192 of WHO´s 193 member states [29]. The 1990s saw a number of articles on outbreaks of measles transmitted in health care settings. Nosocomial transmission of measles is now a widely recognized phenomenon, though different countries have addressed the issue in different ways.

In addition, the ‘80s and ‘90s saw a growing number of publications on outbreaks in schools and universities. The peculiar character of dormitories, which enhance contact between infecteds and susceptibles, can lead to outbreaks in student populations which have received no measles vaccinations, or only one dose.

The 1990s also saw the publication, by the Journal of Infectious Diseases, of a measles outbreak investigation from an athletic event held in a domed stadium [30]. Successive decades have seen publications on outbreaks in public forums, notably the Disneyland measles outbreak of 2015. One ‘90s publication by CDC reported an outbreak of measles among Christian Scientists [31]. This presaged more recent reports on faith-based opposition to vaccination in Africa [32].

The 21st century saw more and more epidemiology publications linked to the newly established global laboratory network, which also provided information on rubella seropositivity among suspected measles cases. (Rubella, which lies outside the scope of this article, is thought to be a likely co-candidate for eradication, once the world community makes a global commitment to measles eradication).

The 21st century also witnessed the changing epidemiology of measles in Africa, with a shift in age distribution of cases towards older age groups [33]. This led to a recent analysis, in this journal, of the impact on measles of wide age-range campaigns [34]. Not surprisingly, wide age-range campaigns are more effective than under-five campaigns in reducing the number and proportion of measles cases in older age groups.

In Europe, the new century saw several reports on measles in anthroposophical communities. More and more, the results of outbreak investigations have brought social scientists into collaboration on the root causes of vaccine hesitancy in those with philosophical or religious objections to vaccination [35]. Two centers of excellence in this growing area are UNICEF and the London School of Hygiene and Tropical Medicine, LSHTM.

An unpublished 2013 report from the UN Foundation summarized operationally important findings from 21st century outbreak reports, as follows: 1) Adult susceptibility when combined with infant susceptibility can contribute significantly to reaching critical thresholds of susceptibility in the population; 2) Several articles focusing on role of health care workers (HCWs), emphasizing importance of addressing susceptibility in this group. 3) In humanitarian emergencies, need aggressive rapid ORI, at times with multiple rounds. 4) Areas around (refugee and IDP) camps also need to be included [36]. The current century has brought into use the expression pockets of susceptibility, in recognition of the persistence of measles in under-vaccinated sub-populations of states and counties of generally high vaccination coverage [37].

As populations of refugees and displaced persons have risen in the present century, so, too, have articles on measles in refugee and IDP camps [38]. Even where coverage levels are high, the population density of camps makes them particularly vulnerable to outbreaks. Recent work has covered the role of seasonality in measles transmission [39]. Since transmission patterns vary in different countries, this work has not yet led to global recommendations on how best to deal with the seasonality of measles transmission.

The current century has seen more and more detailed surveillance of adverse events following immunization (AEFI), both in developed and developing countries. Such surveillance serves not only to quantify the importance of AEFI, but also, in rare cases, to trigger corrective measures when clusters of AEFI cases are found, either during routine or campaign vaccination. As China approaches elimination, that country is using case-control studies to identify risk factors for measles infection [40]. A consistent finding is that contact with clinical services is a risk factor for measles. This points to nosocomial infection as a likely driver of measles perpetuation in the areas studied.

#### Subacute sclerosing panencephalitis

SSPE, a disabling and often lethal sequel of measles, was first described in 1950. A Lancet article of 1967 by Connolly and colleagues, Measles-virus antibody and antigen in subacute sclerosing panencephalitis,” established the link between measles and SSPE [41]. By 1969, Katz was able to answer, in the New England Journal of Medicine, the question How does measles virus cause subacute sclerosing panencephalitis? [42].

Remarkably, succeeding decades, while witnessing progressive declines in SSPE incidence and mortality, have seen a large and growing literature on clinical and virological aspects of the disease. The ‘60s saw only 40 SSPE citations indexed. Since 2010, amid declining incidence, there have been 170 publications indexed on SSPE. Discussion articles in the current decade have been entitled, for example, subacute sclerosing panencephalitis (SSPE): The story of a vanishing disease [43].”.

Since the disease is increasingly rare, it is not surprising that much of the literature is based on individual case reports. Nonetheless, some authors have synthesized existing knowledge about the disease, its etiology and treatment, in review articles [44]. Given the rarity of SSPE and its long latent period, it is not surprising that the disease is not widely known to the general public. This helps to explain why, in many countries, measles is erroneously dismissed as a maladie banale. No student of SSPE would make such a statement.

#### Supplementary immunization activities and routine immunization

Almost all measles vaccinations are administered either by routine immunizations (given through health facilities, outreach, and mobile teams) or by supplementary immunization activities. The Measles & Rubella Initiative, like the Global Polio Eradication Initiative before it, has placed great technical and financial resources into SIAs. These are intended, primarily in developing countries, both to raise the level of community protection in endemic countries and to provide the second dose of vaccine, which is now regarded as essential to interrupting transmission. The ‘60s, ‘70s and ‘80s saw publication of mostly descriptive articles on both routine immunization and SIAs (known then as vaccination campaigns). There were many publications on measles/smallpox campaigns from West Africa (the term SIA was not yet in use). These are primarily of historical interest.

Starting in 1985, the Pan American Health Organization, through its *EPI Newsletter,* documented National Immunization Days (PAHO parlance for SIAs) in support of regional efforts to eliminate polio and measles. In the same decade, UNICEF was supporting multi-antigen vaccination campaigns in support of UCI (universal childhood immunization) with a target date of 1990 to achieve global coverage of 80 percent for the basic 6 vaccinations (diphtheria, pertussis, tetanus, measles, polio, BCG). Those experiences were almost entirely documented in donor reports and internal documents. That decade also saw a multi-country effort by UNICEF to reach “UCI (universal childhood immunization) 1990. In the 1980s, UNICEF spent some funds on support to routine immunization but made large outlays on multi-antigen campaigns whose aim was to achieve rapid increases in coverage for the basic 6 vaccinations and, in some cases, tetanus for women of child bearing age. Almost all of the UNICEF reports were internal or to the donors and the UNICEF Board.

The 1990s saw 55 publications on measles and multi-antigen vaccination campaigns, especially from Latin America, UK, Italy and South Africa. Authors were deeply divided in their opinions as to whether the campaigns were a wise use of resources. One article from the *South African Medical Journal asked The winter 1996 mass immunisation campaign--is it the best strategy for South Africa at this time?* [45].

There were several factors which militated in favor of the SIA approach - initially for polio, then for measles: 1) The success of the Americas in eliminating polio largely through use of SIAs, while polio remained endemic in four of the five other WHO regions, which relied on routine immunization (routine immunization coverage being inadequate, outside Europe, to stop polio transmission). 2) The support of Rotary International for the SIA approach to polio eradication; 3) The decision of WHO, after the 1988 polio eradication commitment, to invest heavily in OPV SIAs in the countries still endemic for polio [46]; 4) The successful experience of PAHO in clearing Latin America and the Caribbean of measles, using the SIA approach.

By the year 2000, the target date for polio eradication had been missed, largely because Asia and Africa lacked the health care services, which, in Latin America, had assured high routine coverage. The RED approach (Reaching Every District), launched in 2002, sought to right this balance by a five-pronged approach to routine immunization [47]. The locus classicus for the RED approach is Reaching every District (RED) approach: a way to improve immunization performance, published by WHO in 2008 and cited 24 times elsewhere [48].

A 2010 published evaluation of RED in the African region found evidence of improvement in delivery of routine immunization services [49]. As of this writing, the World Health Organization´s African Regional Office has prepared revised RED guidelines. The new AFRO guidelines, published in 2018 and place more emphasis on equity, which has been an emphasis in agency and government thinking since the turn of the century. UNICEF, among other agencies, has used quintile analysis to measure differences in coverage among socio-economic strata. Closely related to the RED approach is the interagency GRISP approach (Global Routine Immunization Strategies and Practices), published by WHO.

Remarkably, the present century has seen only 23 published articles on measles SIAs. This probably reflects the predominance of SIAs in the grey literature, including PowerPoint presentations made at EPI meetings. With that said, the dearth of published documentation on measles SIAs limits the readership of the very extensive literature on this subject.

##### The Either-Or dilemma

As long as many countries lack the infrastructure to deliver vaccinations without SIAs, SIAs will continue. With huge expenditures made on SIAs and, more recently, on Immunization Services Strengthening by GAVI and other partners, many authors have examined such issues as the extent to which the SIA approach can better support routine immunization, and the extent to which SIAs reach children who are missed by routine immunization. Several recent articles have explored these issues [50,51]. WHO has recently published guidelines on the conduct of SIAs, including such items as better microplanning and preparedness assessments [52].

A 2016 Cochrane Reviews covered interventions that will increase and sustain the uptake of vaccines in low- and middle-income countries [7]. In their summary, the Cochrane reviewers found evidence for the following interventions: 1) **Giving information and discussing vaccination with parents and other community members at village meetings or at home** probably leads to more children receiving three doses of diphtheria-tetanus-pertussis vaccine (moderate-certainty evidence). 2) **Giving information to parents about the importance of vaccinations during visits to health clinics combined with a specially designed participant reminder card and integration of vaccination services with other health services** may improve the uptake of three doses of diphtheria-tetanus-pertussis vaccine (low-certainty evidence); 3) **Offering money to parents on the condition that they vaccinate their children** may make little or no difference to the number of children that are fully vaccinated (low-certainty evidence); 4 **Using vaccination outreach teams to offer vaccination to villages** on fixed times monthly may improve coverage for full vaccination (low-certainty evidence). The Cochrane reviewers called for more and better randomized controlled trials to improve information on interventions in favor of routine immunization.

#### Progress in measles control and elimination

Although few in number, publications examining measles progress were broad in content in the early decades. In the 1960s, publications not only examined progress in controlling and vaccinating against measles but also progress in eradicating the virus at the country level [53,54]. Though picked up by this article´s search for eradication, not progress, the first indexed publication discussing progress at the global-level occurred in a 1982 article in *The Lancet* [55]. While the 1970s produced only three articles examining measles progress, a dip from the prior decade, two of these articles referenced progress in measles immunization alongside rubella immunization [56,57]. Later, WHO highlighted progress in prevention of measles and rubella in an important 2005 article [58].

Publications on progress jumped in the current century, quite possibly a result of the WHO´s 1998 creation of the Joint Reporting Form (JRF) [59]. Changes in global immunization policy brought about a variety of new ways to measure measles progress. September of 2000 saw the signing of the United Nations´ Millennium Development Goals (MDGs) [60], and several articles subsequently appeared in the decade examining measles progress in the context of the goals [61–63]. MDG4, specifically, called for a reduction in under-five mortality [64]; subsequently, the decade produced indexed articles measuring measles-related mortality reduction at regional and global levels [65,66]. National policy, too, appears to have affected measures of progress. In 1989, the Advisory Committee on Immunization Practices (ACIP) issued an official recommendation for the implementation of a two-dose measles regime in the United States [67]. The year 2004 saw a noteworthy article in *The Journal of Infectious Diseases* investigating progress toward implementation of a second-dose measles immunization requirement for all schoolchildren in the United States [68].

Publication frequency on measles progress has remained high in the current decade, with articles examining progress at scales ranging from city to world [69, 70]. The 2010s have seen continued references to mortality reduction and the Millennium Development Goals [71,72]. As in the 2000s, changes in policy designations have resulted in new measures of progress. W.H.O. Africa´s creation of a “pre-elimination” goal [73], a benchmark towards complete measles elimination, has resulted in a number of publications tracking measles pre-elimination progress in the African Region [74,75]. Additionally, the decade has witnessed discussion of post-elimination progress [76]. It remains to be seen if more such articles are published as measles incidence declines.

#### Mathematical modeling of measles

First indexed in 1973, publications on the mathematical modeling of measles rapidly increased from the 1970s to the present decade, with a modest plateau at the turn of the century. The discussion which follows will focus primarily on those modeling publications which have direct implications for vaccination policy. Of the 13 articles published in the 1980s, nearly one-third specifically examined age-structured models. This topic remains of critical importance, since, with the growth of under-five SIAs, measles in older age groups will play an increasingly important role in measles transmission and in eradication planning. Other articles of the decade were ahead of their time: a 1984 article published in the *Journal of Theoretical Biology*, for instance, examined seasonality in modeling [77]. Additionally, although the measles vaccine had only been in existence for two decades, a 1984 article in the *American Journal of Epidemiology* modeled measles in high-vaccination settings [78].

Though modelling of viral persistence was indexed in the ‘80s, the topic received greater attention in the ‘90s, which saw a tripling of citations on modeling of measles. Additionally, more than in years past, modelling articles of the ‘90s were tied directly to applications, such as assessing economic benefits, evaluating existing vaccination techniques, and crafting immunization policy. These topics continued to be discussed through the 2000s. From the 2000s to the 2010s, publications on measles modeling jumped from 48 to 74. Among modeling studies, some topics may prove to be of interest to policy makers, such as the following: 1) Dynamic transmission models for measles and rubella risk and policy analysis; 2) Modeling of measles transmission to support eradication investment cases; 3) Modeling the impact of HIV infection on measles; 4) Modeling to determine whether mortality reduction goals have been achieved; 5) Modeling to determine the impact of population decline on the dynamics of measles; 6) Modeling the impact of waning immunity; 7) Modeling the impact of vaccination campaigns.

#### Studies of costing and economics

Early costing and economic studies were largely global, often focusing on both the economic cost of the virus and benefit/cost analyses of vaccination programmes within different countries. As early as 1970, a JAMA article investigated the economic worth of the implementation of the immunization surveillance programme in terms of costs to parents in Rhode Island [79].

Articles indexed on economic and costing topics greatly increased from the ‘70s to the ‘80s. Frequency of publication more than doubled in this period. As publication of measles-related costing and economic studies increased, the breadth of the discussion widened. The 1980s saw publications on non-monetary as well as monetary costs (nutritional and energy costs of disease, for instance).

Publications on the subject steadily increased over the next two decades. The 1990s brought a number of articles on cost analyses of immunizing health workers [80–82]. A reference on this subject appeared as early as 1985 [83]. Economic evaluations of two doses of measles vaccine also featured in the decade [84–86]. In the 2000s, a number of articles indexed on the subject discussed the economics of integrated campaigns (e.g., combined campaigns for distributing bed nets while administering measles vaccines) and of supplementary immunization activities [87–90].

In the present decade, publication on economics and costing studies spiked, rising from 30 in the 2000s to 67 in the period 2010 to mid-2018. As MRI expenditures now exceed $50 million annually in agency outlays alone, costing and economics studies have become necessary, both to provide programme managers with the tools for optimal resource allocation and to persuade finance ministries that measles vaccination is bang for the buck. While evaluations of vaccination programmes, supplemental immunizations, and specific outbreaks continue to appear, economic analyses are applied to new tools and challenges in the field. For instance, the current decade has seen studies examining the economic impact of vaccine hesitancy and exemptions [91,92]. The present decade also provides economic evaluations of advances in technology, such as the number of doses per vial, microneedle patches and lab procedures [93–95].

Research on the costs of investigation and contact tracing have been made from developed countries, all showing the expenses associated with measles surveillance [96]. In the US, with the increasing rarity of locally contracted cases, attention has turned to the costs of detection and response to imported measles cases [97]. An important review article estimates the annual costs of measles control at $2.3 billion [98]. Such costing studies lend weight to the arguments in favor of a time-limited global eradication effort..

## Discussion

As the expensive GPEI goes into its fourth decade, the relative costs of time limited eradication and long-term control have been increasingly discussed in the literature. The seminal work of Barrett, cited in 14 other articles, makes the case for time limited eradication [99]. Thompson and Badizadegan contrast the costs of high control of measles over a long time period with the costs of time limited eradication. They conclude, like Barrett, that eradication is the better buy [100]. The work of such authors as Barrett and Thompson addresses the question of whether the world should decide to move towards global eradication. Once that decision is made, through a resolution of the World Health Assembly, costing studies may shift their focus to how best to achieve eradication using different strategies.

### 

#### Autism

Although not discussed in the literature until the late 1990s, publications on measles and autism jumped in the last two years of that decade and substantially increased through the next. While trends for the remainder of the present decade remain to be seen, it appears that publication on this topic has fallen in the 2010s. The first currently indexed article on measles and autism was published in 1998 in the BMJ [101]. This article examines a putative causal link between MMR and autism, first proposed in an article published in The Lancet earlier that year [102] (The Lancet article was subsequently retracted by the editors in 2010 [103]). Thirty-one other articles on measles and autism were indexed by the turn of the century.

In the 2000s, 166 articles were indexed on measles and autism, propelling autism to the most frequently published topic of the decade. Autism-related articles in the 2000s illustrate scientists´ attempts to evaluate the proposed link. Many articles denounced outright the causal link proposed by the 1998 article in The Lancet [104]. In 2001, the Institute of Medicine (IOM) published a report by its Immunization Safety Review Committee concluding that there existed insufficient evidence to assert a causal relationship between the MMR vaccination and the disease; however, the Committee also called for further research [105]. Later, in 2004, the IOM issued another report declaring the absence of a link between autism and the measles-mumps-rubella (MMR) vaccine or the vaccine preservative thimerosal [106].

In 2008, the journal Pediatrics published a paper suggesting that media attention to the controversy had little impact on vaccination rates in the United States [107]. Additional articles published in the present decade continued to investigate whether public discussion of the controversy affected vaccination coverage [108, 109]. While interest in autism appears to have declined, with only 50 articles thus far cited in the present decade, the purported relation to MMR vaccination continues to affect the public. In 2013, an outbreak of measles among pre-teens and teens in Wales is thought to have been the result of autism-related vaccine hesitancy, as was a 2017 measles outbreak among Somali-Americans in Minnesota [110, 111].

### Perspectives for future publications

All six of the WHO regions have created time-limited objectives for regional measles elimination. The next logical step would be a resolution by the World Health Assembly in favor of a time-limited eradication effort against measles alone or measles and rubella. Such a WHA resolution is unlikely to predate the current 2022 end date for the Global Polio Eradication Initiative. Discussions on elimination and eradication renew the perennial debate between advocates of integrated approaches and advocates of a vertical approach. Goodson and colleagues have proposed a diagonal approach to measles and rubella elimination based on lessons learned from polio eradication [23]. Biellik and Orenstein have pointed out that measles-rubella elimination can, when properly implemented, strengthen routine immunization [112]. The conflict between elimination initiatives and integrated approaches is, in the view of some authors, an apparent rather than a genuine conflict [24]. Goodson and colleagues propose the following:

“Focusing efforts on MR elimination after achieving polio eradication would make a permanent impact on reducing child mortality but should be done through a ‘diagonal approach´ of using measles disease transmission to identify areas possibly susceptible to other vaccine-preventable diseases and to strengthen the overall immunization and health systems to achieve disease-specific goals”.

Such an approach, neither vertical nor integrated, would simultaneously serve to stop transmission and to strengthen other components of the Expanded Programme on Immunization. If the next few years see a global commitment to measles eradication, we could expect to see more published research in the following areas: 1) Improvements in the quality of case-based surveillance; 2) Vaccine hesitancy on religious and other grounds; 3) Shifts in age distribution towards adolescents and adults; 4) Sero-surveys as an SIA planning tool; 5) Better predictive models for timing of SIAs; 6) Better understanding of seasonality; 7) Transition of polio surveillance assets to integrated disease surveillance; 8) Wider use of electronics and softwares in epidemiology; 9) Nosocomial transmission and means of combatting it; 10) Epidemiology and economics, with a view to costing measles eradication under different short-term and medium-term scenarios, with or without heavy investments in immunization services strengthening; 11) Combining measles and rubella in a single global eradication initiative; 12) Use of micropatch vaccinations for measles and MR; 13) Methods for identifying high risk districts and localities for pre-emptive vaccination between successive campaigns.

## Conclusion

As of this writing, the most recent lists of research topics are those identified at a WHO meeting held at CDC in 2012 [113] and the following list, prepared for the SAGE (Strategic Advisory Group of Experts in 2014, and reproduced in Orenstein et al. [25]: 1) Strategies to increase coverage in difficult populations;2) Novel strategies to increase vaccine coverage; 3) Strategies to address confidence gaps; 4) Outbreaks in settings with high coverage; 5) Optimal age of measles vaccination; 6) Reasons for low confidence in vaccines; 7) Outbreak response strategies; 8) Strengthen routine immunization & surveillance; 9) Susceptibility profiles to measles and rubella; 10) Measures of vaccine coverage; 11) Epidemiology and surveillance for measles & CRS; 12) Point of care diagnostics. Now that MCV2 vaccination has become widespread, it may be time to find out at what level of coverage governments can safely introduce a four-year interval between SIAs without risk of outbreaks.

### What is known about this topic

- Since the licensing of the first measles vaccine, there has been an increase in published articles on measles and on measles vaccination;
- The topic has attracted authors from many disciplines, notably clinicians, epidemiologists, biostatisticians, mathematical modelers and social scientists;
- The research agenda for measles deserves careful attention as all six regions of the WHO have targeted measles for elimination.

### What this study adds

- This study quantifies the growth of measles publications, decade by decade. The number of index publications on measles has more than doubled since the 1960s;
- This study quantifies trends in measles publications, decade by decade, with special emphasis on the current century;
- This study summarizes several of the most recent reviews of future research priorities on measles, as proposed by specialists in the field.

## Competing interests

The authors declare no competing interests.
